# Decreased level of soluble receptor activator of nuclear factor-κβ ligand (sRANKL) in overweight and obese children

**DOI:** 10.3389/fendo.2022.963467

**Published:** 2022-08-19

**Authors:** Michał Erazmus, Małgorzata Rumińska, Ewelina Witkowska-Sędek, Anna M. Kucharska, Anna Stelmaszczyk-Emmel, Anna Majcher, Beata Pyrżak

**Affiliations:** ^1^ Department of Paediatrics and Endocrinology, Medical University of Warsaw, Warsaw, Poland; ^2^ Department of Laboratory Diagnostics and Clinical Immunology of Developmental Age, Medical University of Warsaw, Warsaw, Poland

**Keywords:** sRANKL, OPG/sRANKL ratio, cardiovascular risk factors, obesity, children

## Abstract

**Introduction:**

Childhood obesity contributes to the development of cardiovascular diseases. The molecular pathway – receptor activator of nuclear factor-κβ ligand (RANKL), its receptor RANK and osteoprotegerin (OPG) - takes part not only in bone metabolism but is also involved in the atherosclerosis process. RANKL stimulates osteogenic differentiation and calcification of vascular smooth cells. The associations between the OPG-sRANKL system and various cardiovascular risk factors were displayed. We aimed to evaluate the relationships between serum sRANKL (soluble RANKL) levels and the OPG/sRANKL ratio with cardiometabolic risk factors in overweight and obese children.

**Material and methods:**

The study included 70 children with overweight and obesity (mean age 13.0 ± 2.8) and 35 age-matched normal weight, healthy peers as a control group. In all patients, anthropometric measurements and laboratory tests were performed. Additionally, an oral glucose tolerance test (OGTT) was made only in overweight and obese children. Atherogenic and insulin resistance indices were calculated.

**Results:**

Overweight and obese children had lower sRANKL levels compared to the control group (median 276.95 vs 325.90, p=0.011), and consequently a higher OPG/sRANKL ratio (0.02 vs 0.01, p = 0.013). The studied children in the lowest quartile of sRANKL levels had higher body weight, Body Mass Index, waist circumference and increased glucose and insulin levels 60 minutes after OGTT and higher uric acid values compared to children in the highest quartile. In multivariable linear regression analysis sRANKL negatively correlated only with uric acid (β = - 0.508, p = 0.041). No association was found for the OPG/sRANKL ratio.

**Conclusion:**

Excess fat mass seems to alter the OPG/RANKL ratio mainly by reducing serum sRANKL levels. The correlation between sRANKL and uric acid may suggest a contribution of the OPG-sRANKL system in the cardiometabolic process, but that observation should be confirmed in future studies.

## Introduction

Overweight and obesity in childhood are associated with increased mortality due to cardiovascular (CV) diseases in adulthood. The atherosclerosis process has long been known to start at an early age and is linked to obesity and disorders related to excess fat mass (1, 2). The combination of classic CV risk factors such as carbohydrate-lipid metabolic abnormalities, insulin resistance, hypertension, as well as altered concentrations of bioactive adipocytokines and adhesion molecules and following immune reactions or chronic inflammation appears to explain at least a part of the relationship of adiposity with endothelial dysfunction (1, 3–5). Nevertheless, the mechanisms linking early fat mass accumulation with the atherosclerosis process are still poorly understood. Recent studies suggested that the cytokine pathway of the OPG/RANKL/RANK (Osteoprotegerin/receptor activator of nuclear factor- kβ ligand/receptor activator of nuclear factor kβ) is implicated in vascular calcification, which is linked with bone metabolism (6, 7). In the bone, RANKL expressed on the surface of osteoblasts (OB) and bone marrow stromal cells (BMSC) initiates differentiation, maturation, and activity of osteoclasts after binding to its receptor (7–11). OPG acts as a soluble decoy receptor, which inhibits osteoclastogenesis after binding with RANKL. OPG is also expressed in endothelial cells (ECs) and vascular smooth muscle cells (VSMCs), whereas RANKL is mainly expressed in infiltrating T cells and activated ECs (12, 13). Several bone structural proteins were identified in calcified vascular tissue in the experimental study (9, 14). It is suggested, that OPG protects against the RANKL-RANK induced bone resorption and vascular calcification. So, the relative balance between OPG and RANKL may affect bone metabolism but also the immune and vascular system. It seems that the ratio of these factors better reflects CV risk (15–17).

Clinical studies conducted mainly in adults supported the association between the OPG-RANKL system and CV pathology. The higher serum levels of OPG correlated with multiple cardiometabolic risk factors as well as with advanced atherosclerosis, CV incidents, and mortality or morbidity (18–20). Several studies show an association of the serum soluble RANKL (sRANKL) level with Body Mass Index (BMI), lipids profile, Homeostatic Model Assessment- Insulin Resistance (HOMA-IR), diabetes, blood pressure, C-reactive protein (CRP) (19, 21–24), carotid intima-media thickness (cIMT) (25) and CV events (26, 27).

In the previous study (28) investigating the relationships between the serum OPG concentrations and chosen cardiometabolic risk factors in overweight and obese children and adolescents, we found that children with excess fat mass had decreased concentration of sRANKL and consequently increased the OPG/sRANKL ratio compare to their normal-weight peers. OPG levels in blood serum were comparable between these groups. Therefore in the present study, we focused on the analysis of the serum sRANKL and the OPG/sRANKL ratio in the same group of patients. Given the above-mentioned clinical studies in adults, we hypothesized, that sRANKL or the OPG/sRANKL ratio correlates with typical cardiometabolic risk factors. Research on this topic in the pediatric population is limited.

## Material and methods

The study was carried out at the Department of Pediatrics and Endocrinology Medical University of Warsaw. The protocol was approved by the Bioethical Committee of the Medical University of Warsaw in accordance with the Declaration of Helsinki.

The study group consisted of 70 children and adolescents (36 boys, 34 girls) with overweight (n = 17) and obesity (n = 53), the ages of 7.0 to 17.8 years. Overweight and obesity were defined according to the value of z-score BMI for age and sex: z-score BMI ≥ 1 was considered overweight, z-score BMI ≥ 2 obese (29). Overweight and obese in the group of children enrolled in this study were caused by incorrect eating habits. Exclusion criteria were genetic and endocrine causes of excess fat mass, as well as severe chronic diseases such as diabetes, hypertension, and hepatic or renal disturbances. The history of taking any medications, including vitamin D was negative.

The control group consisted of 35 healthy, age- and sex-matched children and adolescents (21 boys, 14 girls). Their physical parameters were within the normal range.

### Anthropometric measurements

In all participants, physical examinations were performed. Height (cm), weight (kg), waist and hip circumferences (cm), and the thickness of skinfolds under the triceps brachii muscle and under the inferior scapular angle (mm) were measured by a qualified anthropologist. Based on the obtained results, BMI, waist-to-hip ratio (WHR), waist-to-height ratio (WHtR) as well as the percentage of fat mass (%BFM) from the sum of 2 skinfolds using the Slaughter formula were calculated (30). The degree of excess fat was expressed as z-score BMI (SDS BMI, standard deviation score), calculated using the LMS (lambda, mu, sigma) method to normalize the data for the age and sex using polish reference values (31, 32).

### Laboratory tests

In all patients, blood samples were taken after night fasting. Both OPG (pmol/l) and the total sRANKL (pmol/l) concentrations in blood serum were determined by an enzyme immunoassay - ELISA test (DRG Instruments GmbH, Germany). Lipids profile: total cholesterol (TC, mg/dl), high-density lipoprotein cholesterol (HDL-C, mg/dl), and triglyceride (TG, mg/dl) were measured using the colorimetric enzymatic method using a VITROS 5600 Chemistry Analyzer (Ortho-Clinical Diagnostics, New Jersey, USA). Low-density lipoprotein cholesterol concentration (LDL-C, mg/dl) was calculated using the Friedewald formula (33). Fasting glucose (mg/dl) and insulin (µIU/ml) concentrations and additionally, oral glucose tolerance test (OGTT, oral glucose load of 1.75 g/kg body weight up to the maximum of 75 g) only in the group of overweight and obese children were measured by glucose oxidase colorimetric method using VITROS 5600 Chemistry Analyzer and immunoassay method using IMMULITE 2000 Xpi Analyzer (Siemens, Erlangen, Germany), respectively. The concentrations of glycosylated hemoglobin (HbA1c, %) were determined by ion-exchange high-performance liquid chromatography (HPLC) using D-10 Hemoglobin Analyzer (BIO-RAD). Subsequent blood tests: uric acid (UA, mg/dl), calcium (Ca, mg/dl), phosphorus (P, mg/dl) levels, and total alkaline phosphatase ***(*** ALP, U/L) activity were measured by the dry chemistry method using VITROS 5600 Chemistry Analyzer). On the same analyzer, CRP (mg/dl) concentrations were measured using the fixed-point immune-rate method. The serum concentration of intact parathyroid hormone **(**PTH, pg/ml) and the 25-hydroxyvitamin D (25(OH)D, ng/ml) were measured by immunoassay method using an IMMULITE 2000 Xpi Analyzer and Architect Analyzer (Abbott Diagnostics; Abbott Park, IL), respectively.

The obtained serum results were used to calculate the insulin resistance (HOMA-IR, QUICKI - Quantitative Insulin Sensitivity Check Index, Matsuda index) and atherogenic (non-HDL, TG/HDL-C ratio) indices (34–37).

In overweight and obese children calcium, phosphorus, and creatinine excretion were measured in 24-hour urine samples by dry chemistry system using VITROS 5600 Chemistry Analyzer and were converted to mg/kg/24 hours. Tubular Reabsorption of Phosphate (TRP) was calculated (http://www.scymed.com/en/smnxps/pshpd274.htm).

### Statistical analysis

Statistical calculations were performed using the SPPS 13.3 software. To check the normality of data distribution the Shapiro-Wilk test was used. The data with normal distribution were presented as mean and standard deviation (SD), the data with non–normal distribution as median with interquartile range (IQR). The study group was compared to the control group by using a Student’s T-test or U Mann*-*Whitney test. Moreover, we compared the distribution of anthropometric and biochemical parameters in overweight and obese children after stratification according to the sRANKL quartiles and the OPG/sRANKL quartiles. For the comparison of more than three groups, One-Way Analysis of Variance (ANOVA) or a Kruskal-Wallis test were used, as appropriate. To provide detailed information regarding the differences among various combinations of groups stratified according to quartiles Tukey *post-hoc* tests with Bonferroni corrections were made for the ANOVA test and the Dunn *post-hoc* tests with Bonferroni corrections for the Kruskal-Wallis test. The association between two ranked variables was measured by using the Spearman correlation coefficient test. To evaluate independent relationships between sRANKL and the OPG/sRANKL ratio, which were considered dependent variables, and selected anthropometric and biochemical parameters, which were considered independent variables, the multiple linear analysis was used. The p-value < 0.05 was considered statistically significant.

## Results

The circulating sRANKL levels in children with overweight and obesity were significantly lower in comparison to the control group (median (IQR) = 261.36 (168.66); 283.28 (238.55); 325.90 (247.30), p = 0.019; p = 0.029, respectively). The sRANKL concentrations did not differ between overweight and obese children (p = 0.473). The median (IQR) of the OPG concentrations were comparable between children with overweight (3.84 (2.23)) and obesity (3.47 (1.28)) and their normal peers (3.74 (1.58)). So, the overweight and obese children were taken together (consider as the study group) for further analysis.

The comparison of anthropometric measurements and cardiometabolic parameters in serum blood as well as calcium-phosphorus metabolism parameters in blood and in urine in the study group and in the control group we presented in **Table 1**.

**Table 1:** The comparison of anthropometric measurements, OPG, sRANKL, and biochemical parameters between overweight and obese children and their peer with normal weight.

Variables	Overweight and obese children(n = 70)	Non-obese children(n = 35)	p-value
Anthropometric measurements			
Height (cm)	160.6 ± 14.2	159.8 ± 13.6	Ns
Body weight (kg)	77.0 ± 21.0	50.8 ± 12.7	< 0.001
BMI (kg/m2)	29.4 (5.5)	18.7 (4.6)	< 0.001
BMI SDS	2.1 (0.4)	0.0 (1.1)	< 0.001
WC (cm)	87.9 ± 10.5	64.7 ± 6.2	< 0.001
HC (cm)	103.1 ± 13.0	83.7 ± 9.4	< 0.001
WHR	0.9 ± 0.1	0.8 ± 0.0	< 0.001
WHtR	0.5 ± 0.1	0.4 ± 0.0	< 0.001
% BFM	34.8 (7.4)	26.4 (8.1)	< 0.001
**Blood tests**			
OPG (pmol/l)	3.61 (1.36)	3.74 (1.58)	ns
sRANKL (pmol/l)	276.00 (188.56)	325.90 (247.30)	0.011
OPG/sRANKL ratio	0.02 (0.02)	0.01 (0.01)	0.013
fasting glucose (mg/dl)	85.85 ± 6.45	83.27 ± 6.97	Ns
fasting insulin (µIU/ml)	13.10 (11.77)	8.49 (7.92)	<0.001
HOMA -IR	2.84 (2.62)	1.71 (1.59)	<0.001
QUICKI	0.33 (0.04)	0.35 (0.06)	<0.001
MATSUDA	2.85 (1.82)	-	
TC (mg/dl)	162.08 ± 26.75	153.09 ± 23.77	Ns
HDL-C (mg/dl)	45.65 ± 11.68	62.28 ± 11.97	< 0.001
LDL-C (mg/dl)	93.36 ± 25.51	78.05 ± 19.64	0.013
TG (mg/dl)	104.00 (34.00)	63.00 (18.00)	< 0.001
TG/HDL-C	2.32 (1.85)	1.00 (0.40)	<0.001
non HDL	116.43 ± 26.27	90.81± 20.34	<0.001
UA (mg/dl)	5.82 ± 1.09	4.34 ± 1.17	0.001
Ca (mg/dl)	10.00 (0.35)	9.90 (0.40)	Ns
P (mg/dl)	4.92 ± 0.78	4.60 ± 1.00	Ns
25(OH)D (ng/ml)	17.80 (10.70)	22.80 (7.40)	0.025
ALP (U/L)	170.00 (138.00)	110.00 (133.00)	0.032
PTH (pg/ml)	21.80 (25.80)	20.60 (20.00)	Ns
CRP (mg/dl)	0.5 (0.05)	0.5 (0.0)	Ns
**Urine tests**			
Ca (mg/kg/24 h)	0.79 (0.81)	1.68 (0.85)	*0.085*
P (mg/kg/24 h)	9.47 (4.88)	12.79 (12.69)	Ns
TRP (%)	91.51(1.89)	90.67 (5.12)	Ns

Data are presented as mean ± standard deviation (SD) or median values with interquartile range (IQR) as appropriate.

BMI, body mass index; BMI SDS, body mass index standard deviation score; WC, waist circumference; HC, hip circumference; WHR, waist-to-hip ratio; WHtR, waist-to-height ratio; % BFM, % of body fat mass; OPG, osteoprotegerin; sRANKL, soluble nuclear factor kappa B ligand; HOMA - IR, Homeostasis model assessment for insulin resistance index; QUICKI, quantitative insulin sensitivity check index; TC, total cholesterol; HDL-C, high-density lipoprotein cholesterol; LDL-C, low-density lipoprotein cholesterol; TG, triglycerides; TG/HDL-C, triglycerides to high-density lipoprotein cholesterol ratio; non-HDL, non-high-density lipoprotein cholesterol; UA, uric acid; Ca, calcium; P, phosphorus; 25(OH)D, 25-hydroxy vitamin D; ALP, alkaline phosphatase; PTH, parathyroid hormone; CRP, C-reactive protein; TRP, Tubular Reabsorption of Phosphate; ns, nonsignificant.

As expected, the overweight and obese children had atherogenic lipid profiles, higher insulin resistance status, and increased UA concentrations than their normal peers. Moreover, the studied participants had lower concentrations of 25(OH)D and higher ALP activity. The calcium and phosphorus concentration in blood serum and urine did not differ significantly between the study and control groups (**Table 1**).

### Correlation of sRANKL and the OPG/sRANKL ratio with anthropometric and biochemical parameters in normal weight, overweight and obese children taken together to analysis

In the Spearman correlation coefficient analysis, sRANKL in normal weight, overweight and obese children negatively correlated with body weight (R = - 0.255, p = 0.009), BMI (R = - 0.240, p = 0.014), waist circumference (WC, R = - 0.333, p = 0.003), fasting glucose (R = - 0.197, p = 0.047), UA (R = - 0.388, p = 0.002), and HbA1c (R = - 0.312, p = 0.012). For the OPG/sRANKL ratio we observed association with WC (R = 0.239, p = 0.040), UA (R = 0.326, p = 0.010), and HbA1c (R = 0.425, p = < 0.001).

### Anthropometric and biochemical parameters after stratification according to sRANKL and the OPG/sRANKL ratio quartiles in overweight and obese children

The distribution of chosen anthropometric and biochemical parameters after stratification according to the sRANKL quartiles in overweight and obese children is presented in **Table 2**.

**Table 2:** Characteristics of chosen anthropometric and biochemical parameters after stratification according to the sRANKL quartiles in the study group.

Quartiless RANKL(pmol/l)	Quartile 1 (n=17)< 162.84	Quartile 2 (n=18) 162.84 - 275.99	Quartile 3(n=18) 276.00 - 359.89	Quartile 4 (n=17)≥ 359.90	p-trend
*Variables:*					
Body weight (kg) BMI (kg/m^2^) WC (cm) Glucose 60’in OGTT (mg/dl) Insulin 60’ in OGTT (µIU/ml) UA (mg/dl)	78.6 ± 15.5 29.7 (10.2) 89.8 ± 7.2 138.1 ± 25.9 116.0 (83.3) 6.3 ± 1.2	79.4 ± 23.7 29.8 (12.9) 87.1 ± 10.0 139.2 ± 32.1 96.7 (289.4) 6.0 ± 0.9	80.7 ± 22.3 29.6 (21.7) 91.5 ± 12.3 118.7 ± 32.2 99.0 (281.0) 5.6 ± 1.3	67.9 ± 20.3* 28.0 (21.2)* 82.7 ± 10.3* 116.9 ± 29.6*^+^ 76.4 (214.0)* 5.3 ± 0.7*^+#^	0.256 0.650 0.129 *0.061* *0.097* *0.069*

Data are presented as mean ± standard deviation (SD) or median values with interquartile range (IQR) as appropriate.

The sRANKL, soluble nuclear factor kappa B ligand; BMI, body mass index; WC, waist circumference; OGTT, oral glucose tolerance test; UA, uric acid.

*p < 0.05 found in the comparison of Q1 to Q4.

+p < 0.05 found in the comparison of Q1 to Q3.

#p < 0.05 found in the comparison of Q2 to Q4.

^p < 0.05 found in the comparison of Q2 to Q3.

$p < 0.05 found in the comparison of Q 3 to Q4.

We found that children with the lowest concentrations of sRANKL (first quartile) had higher values of body weight (p = 0.014), BMI (p = 0.029), WC (p = 0.015), as well as increased concentrations of glucose and insulin in 60 minutes of the OGTT (p = 0.014, p = 0.008, respectively) as compared to children with the highest value of sRANKL (fourth quartile). Moreover, we observed, that together with increased quartiles of sRANKL, the concentrations of UA decreased and the differences between the first quartile (Q1), second quartile (Q2), third quartile (Q3), and fourth quartile (Q4) were statistically significant (Q1 vs Q3: p = 0.018, Q1 vs Q4: p = 0.014, Q2 vs Q3: p = 0.053, Q2 vs Q4: p = 0.030).

After dividing the overweight and obese children for subgroups stratification according to the OPG/sRANKL ratio (presented in **Table 3**) we observed that together with increased quartiles of the OPG/sRANKL ratio, HbA1c levels increased (Q1 vs Q2: p = 0.015, Q1 vs Q3: p = 0.032, Q1 vs Q4: p = 0.015). Similar to sRANKL, children with the lowest values of the OPG/sRANKL ratio (Q1) had smaller WC compared to children in the fourth quartile (Q1 vs Q4 p = 0.043) and lower concentration of glucose in 60 minutes of OGTT and UA compared to children in third and fourth quartiles (for glucose: Q1 vs Q3: p = 0.050, Q1 vs Q4: p = 0.036, for UA: Q1 vs Q3: p = 0.046). Moreover, we observed, that LDL-C and TC levels statistical differ between Q2 and Q3 (p = 0.009 and p = 0.016, respectively) and Q2 and Q4 (p = 0.051, p = 0.044).

**Table 3:** Characteristics of chosen anthropometric and biochemical parameters after stratification according to the OPG/sRANKL ratio quartiles in the study group.

Quartiles	Quartile 1(n=17)	Quartile 2(n=18)	Quartile 3(n=18)	Quartile 4(n=17)	p-trend
OPG/sRANKL	< 0.0084	0.0084-0.0157	0.0158-0.0269	≥ 0.0270	
*Variables:* WC (cm) Glucose 60’ in OGTT (mg/dl) TC (mg/dl) LDL-C (mg/dl) UA (mg/dl) HbA1c (%)	85.7 ± 8.1 117.1 ± 31.2 157.3 ± 24.3 89.0 ± 24.5 5.5 ± 0.6 5.1 (1.6)	87.6 ± 12.6 121.7 ± 26.4 151.9 ± 19.4 82.1 ± 21.0 5.5 ± 1.2 5.4 (0.7)	85.2 ± 12.4 138.2 ± 30.4 170.6 ± 24.9 105.2 ± 22.15 6.4 ± 1.4 5.4 (1.0)	92.1 ± 7.3* 140.5 ± 32.5*^+^ 170.1 ± 32.0^#^^ 100.65 ± 28.2^#^^ 6.1 ± 1.0^+^ 5.5 (1.1)*^+^	0.250 *0.076* *0.093* **0.029** *0.067* **0.030**

Data are presented as mean ± standard deviation (SD) or median values with interquartile range (IQR) as appropriate.

The OPG/sRANKL ratio, osteoprotegerin to soluble nuclear factor kappa B ligand ratio; WC, waist circumference; OGTT, oral glucose tolerance test; TC, total cholesterol; LDL-C, low-density lipoprotein cholesterol; UA, uric acid; HbA1c, glycosylated hemoglobin.

*p < 0.05 found in the comparison of Q1 to Q4.

+p < 0.05 found in the comparison of Q1 to Q3.

#p < 0.05 found in the comparison of Q2 to Q4.

^p < 0.05 found in the comparison of Q2 to Q3.

&p < 0.05 found in the comparison of Q3 to Q4.

### Correlation of sRANKL and the OPG/sRANKL ratio with anthropometric and biochemical parameters in overweight and obese children and adolescents

In the Spearman correlation coefficient analysis, serum sRANKL in overweight and obese children were inversely related to fasting glucose and glucose in 60 and 90 minutes of the OGTT (R = - 0.258, p = 0.032; R = - 0.389, p = 0.001; R = - 0.309, p = 0.014, respectively) and insulin in 60 minutes of the OGTT (R = - 0.308, p = 0.014), as well as with UA (R = - 0.387, p = 0.000). The OPG/sRANKL ratio positively correlated with glucose in 60 and 90 minutes of the OGTT (R = 0.356, p = 0.004; R = 0.262, p = 0.041, respectively), insulin in 60 minutes of the OGTT (R = 0.258, p = 0.041), UA (R = 0.326, p = 0.029) and HbA1c (R = 0.3576, p = 0.009). We did not find any association of both sRANKL and the OPG/sRANKL ratio with calcium-phosphorus metabolism parameters.

**Figure 1:**
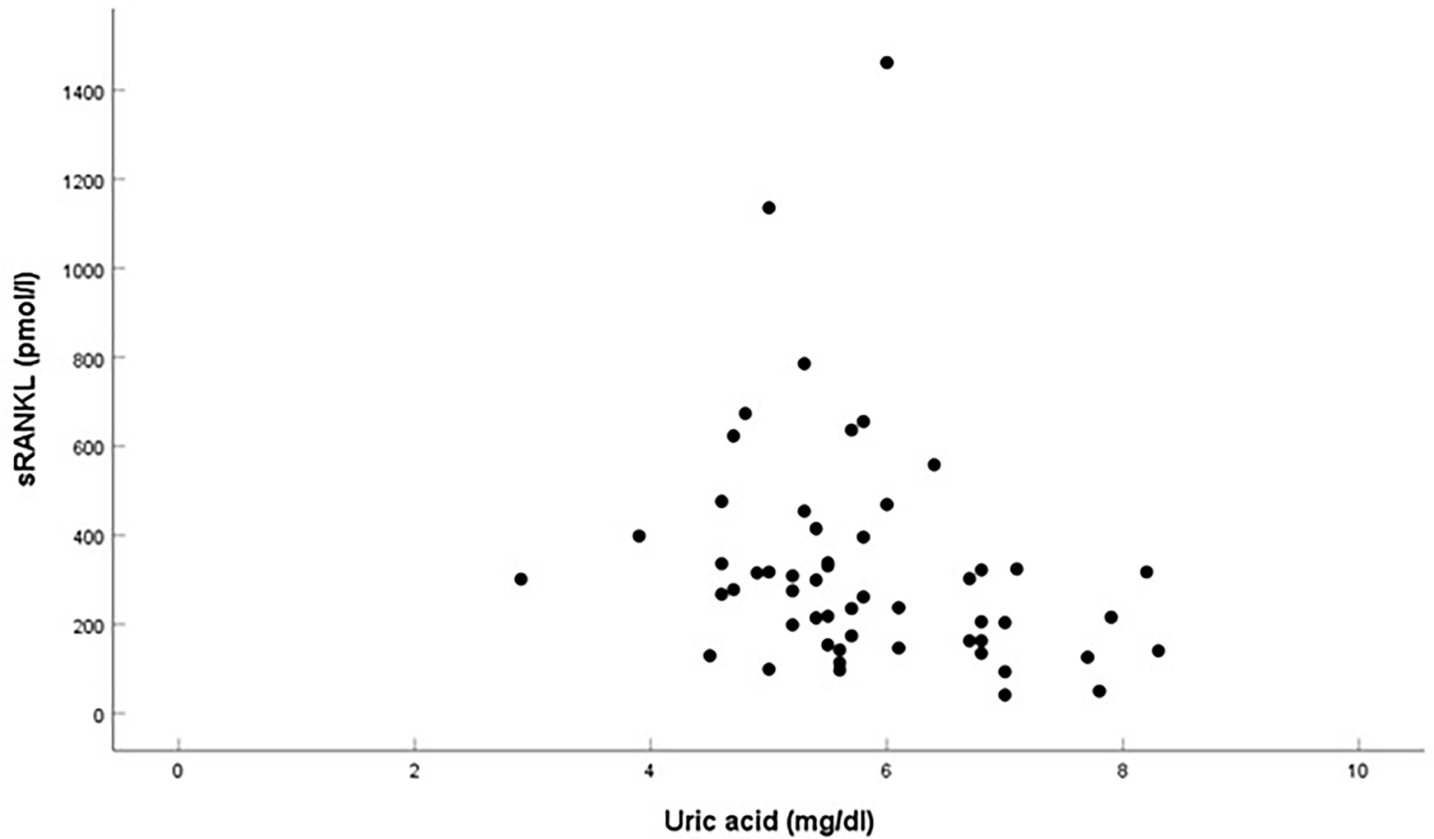
Correlation between sRANKL and urid acid in overweight and obese children.

In multivariable linear regression analysis, where sRANKL was the dependent variable and BMI SDS, WC, HbA1c, HOMA-IR, TC, HDL-C, and LDL-C were the independent variables, sRANKL in overweight and obese children correlated only with UA (β = - 0.508, p = 0.041, 95%CI: -187.74 - -4.12) (**Figure 1**). In a similar model, which was performed for the OPG/sRANKL ratio as the dependent variable, we did not find any association.

### Correlation of UA with anthropometric and biochemical parameters in overweight and obese children and adolescents

In the Spearman correlation coefficient analysis serum UA in overweight and obese children positively correlated with body weight (R = 0.450, p = < 0.001), BMI (R = 0.365, p = 0.006), BMI SDS (R = 0.290, p = 0.033), WC (R = 0.463, p = 0.001), CRP (R = 0.397, p = 0.005), glucose and insulin in 60 minutes of the OGTT (R = 0.306, p = 0.024; R = 0.329, p = 0.015, respectively).

## Discussion

The decreased sRANKL level in our overweight and obese children leads to the imbalance between the levels of the circulating OPG and sRANKL and consequently increases the OPG/sRANKL ratio. In this study, we analyzed whether there is an association between the sRANKL concentration and the OPG/sRANKL ratio with metabolic disturbances related to excess fat mass.

Data regarding the correlation of the OPG-RANKL system with excess fat mass and cardiometabolic risk factors in children and adolescents are limited and inconsistent. In one study (38) involving healthy children, sRANKL levels slightly decline with age but were not related to gender or BMI SDS. While in another study (39) sRANKL concentrations were higher in boys than in girls and increased with age and body weight percentile. In a total scholar population of 600 children between the ages of 6 and 12 years, higher sRANKL levels were found in those with central obesity determined by the WC and negatively correlated with HDL-C (40). In contrast, in another study, sRANKL levels in groups of obese children did not differ compared to the control group and did not correlate with parameters describing nutritional status (41, 42) and with atherogenic and insulin resistance indices (41). Our research has produced different results. We noticed an inverse relationship between the sRANKL concentration and the chosen anthropometric parameters. The subgroup of overweight and obese children with the lowest sRANKL concentration had higher body weight, BMI, and WC than those with the highest sRANKL levels. Abdominal obesity appears to affect the higher OPG/sRANKL ratio, but these dependencies are not consistent directionally across all quartiles. On the other hand, we did not find the differences between sRANKL levels or the OPG/sRANKL ratio and the groups of overweight and obese children, which raises doubts about the effect of adipose tissue on these studied parameters. Conflicted results regarding the association of the sRANKL concentration with fat mass have also been reported in adult studies [positive association with BMI - (21, 22), no relationship - (43, 44)].

In the 3200 Framingham Study adult participants, sRANKL levels displayed inverse association with the multiple CV disease risk factors including diabetes (19). The decreased serum concentration of sRANKL in patients with type 2 diabetes (T2DM) compared to the controls was documented in other studies (20, 23, 45), but not in all (22). Inline, we also found an inverse relationship between sRANKL and glucose concentrations. An increase in the OPG/sRANKL ratio caused an increase in HbA1c concentration. Meanwhile, in children, 13.04 ± 3.53 years of age with type 1 diabetes mellitus (T1DM) both sRANKL and OPG levels were elevated compared to their healthy peers, but these study assessed their relationship with low bone mass (46). In another group of children of similar age and with T1DM both markers did not differ in comparison to the control group, but in those with microalbuminuria, sRANKL negatively correlated with cIMT (25). Clinical trials in adults also yielded different results, both decreased (6, 43) as well as increased (26, 27) sRANKL levels were related to CV diseases. Moreover, Zampetti et al. (47) study showed an association of the OPG/sRANKL ratio with left ventricular hypertrophy and geometric remodeling in overweight in obese boys. Of note, Gaudio et al. (20) supported the role of the increased the OPG/sRANKL ratio as a possible marker of progression of vascular dysfunction. Our overweight and obese children also had an increased OPG/sRANKL ratio. The role of RANKL in the pathogenesis of CV diseases is multifactorial. RANKL enhances chemokine release (monocyte chemoattractant protein-1 (MCP-1)), promotes monocyte/macrophage matrix migration, directly stimulates osteogenic differentiation of VSMC *via* a decrease Matrix Gla Protein (MGP), and indirectly *via* increased BMP-2, as well as increases matrix metalloproteinase activity leads to matrix degeneration (13, 26, 48). Moreover, in the immune and inflammatory pathways promoting atherosclerosis, activated T cells are involved which are a source of RANKL and pro-inflammatory cytokines (such as tumor necrosis factor α (TNF-α), interleukin-1 (IL-1), and interleukin - 6 (IL-6)), that up-regulate RANKL expression (6, 12, 49).

New findings from the present study are the detection of the inverse relationship between UA and sRANKL, after adjustment of lipids and insulin resistance variables. In the literature, the association between elevated serum UA with obesity, insulin resistance, glucose and lipids disturbances, metabolic syndrome, hypertension, carotid atherosclerosis, and an increased incidence of CV events in young adults, is well documented (2, 50–54). Excess of UA has paradoxically pro-oxidant effects in the vascular cells, impaired nitric oxide production, increased cytokines (IL 1β, IL 6, TNF α, CRP, MCP-1), and platelet-derived growth factors expression, leading to endothelial dysfunction and VSMC proliferation (55–57). Similar intracellular oxidative stress, together with inflammatory cytokines induced by UA take part in the pathogenesis of osteoporosis (57). Moreover, monosodium urate crystals increased mRNA expression of the RANKL-induced osteoclast formation in an experimental study (58). So, we speculated that the inflammatory process may be the link between obesity, UA, and sRANKL.

The negative correlations between sRANKL and obesity, and some cardiometabolic parameters are difficult to explain. RANKL exists mainly as a transmembrane protein (cellular form). Its soluble form (sRANKL), which was measured in our study, is only a small fraction of the total amount of this cytokine. It is not clear, what is the impact of the bone or vascular microenvironment on the circulating concentration of both OPG and sRANKL. Maybe these factors exert a paracrine action on the local cells and therefore their serum concentrations do not mirror their true interactions (13). Another explanation is that maybe the changes in the vessels or bone metabolism led to a compensatory increase of OPG and the neutralization of sRANKL giving the decreased sRANKL levels in serum (the circulating OPG bound to its ligands RANKL that is not detected using the ELISA test). Moreover, a wide array of factors regulates RANKL production. As we mentioned above, RANKL expression is stimulated by proinflammatory cytokines, linked to low-grade inflammation related to obesity (26). In Puengel et al. study (45) sRANKL positively correlated with adiponectin, leptin receptor, and ghrelin in critically ill patients. Whereas, in OB and the BMSC RANKL expression is up-regulated also by various pro-resorptive stimuli such as PTH, 1,25-dihydroxyvitamin D3, steroids, prostaglandin E2 (11, 12, 57). Despite higher CRP concentration in overweight and obese children compared to their normal peers, this inflammatory marker did not correlate with sRANKL. Except that, PTH concentrations did not differ between groups. Moreover, we have no knowledge of bone metabolism in our children. Higher total ALP activity was found in our overweight and obese patients, making clinical interpretation difficult without fractionation of these ALP isoforms (59, 60). Serum ALP may be associated with vascular calcification, while bone-specific ALP (BAP) is a marker of bone formation (59). No assessment of BAP and bone mineral density (BMD) is a major limitation of our study.

## Conclusion

Excess fat mass seems to alter the OPG/RANKL ratio mainly by reducing serum sRANKL levels. The correlation between sRANKL and UA may suggest the participation of the OPG-sRANKL system in the cardiometabolic process, but that observation should be confirmed in future studies.

## Data availability statement

The original contributions presented in the study are included in the article/supplementary material. Further inquiries can be directed to the corresponding author.

## Ethics statement

The studies involving human participants were reviewed and approved by Medical University of Warsaw. Written informed consent to participate in this study was provided by the participants’ legal guardian/next of kin. Written informed consent was obtained from the individual(s), and minor(s)’ legal guardian/next of kin, for the publication of any potentially identifiable images or data included in this article.

## Author contributions

ME wrote the manuscript and collected the literature data. MR designed the study, wrote the manuscript and prepared tables and figures, and collected the literature data. EW-S wrote the manuscript and collected the literature data. AK - critical review of the article. AS-E made the laboratory tests and interpreted the results. AM made the anthropometric parameters and interpreted the results. BP - a critical review of the article. All authors have read and approved the manuscript.

## Conflict of interest

The authors declare that the research was conducted in the absence of any commercial or financial relationships that could be construed as a potential conflict of interest.

## Publisher’s note

All claims expressed in this article are solely those of the authors and do not necessarily represent those of their affiliated organizations, or those of the publisher, the editors and the reviewers. Any product that may be evaluated in this article, or claim that may be made by its manufacturer, is not guaranteed or endorsed by the publisher.
